# SARS-Cov-2 Natural Infection in a Symptomatic Cat: Diagnostic, Clinical and Medical Management in a One Health Vision

**DOI:** 10.3390/ani11061640

**Published:** 2021-06-01

**Authors:** Alda Natale, Elisa Mazzotta, Nicoletta Mason, Letizia Ceglie, Monica Mion, Annalisa Stefani, Alice Fincato, Francesco Bonfante, Alessio Bortolami, Isabella Monne, Laura Bellinati, Carmine Guadagno, Erika Quaranta, Ambra Pastori, Calogero Terregino

**Affiliations:** 1 Istituto Zooprofilattico Sperimentale delle Venezie, 35020 Legnaro, Italy; elisa.mazzotta@unipd.it (E.M.); lceglie@izsvenezie.it (L.C.); mmion@izsvenezie.it (M.M.); astefani@izsvenezie.it (A.S.); afincato@izsvenezie.it (A.F.); fbonfante@izsvenezie.it (F.B.); abortolami@izsvenezie.it (A.B.); imonne@izsvenezie.it (I.M.); lbellinati@izsvenezie.it (L.B.); equaranta@izsvenezie.it (E.Q.); apastori@izsvenezie.it (A.P.); cterregino@izsvenezie.it (C.T.); 2 Department of Animal Medicine, Productions and Health (MAPS), University of Padova, 35020 Legnaro, Italy; 3 Veterinarian, Clinician, Marcon, 30020 Venezia, Italy; nicoletta.mason@virgilio.it; 4 ULSS 3 Serenissima, 30135 Venezia, Italy; carmine.guadagno@aulss3.veneto.it

**Keywords:** SARS-CoV-2, cat, medical features and management, serology, RT-qPCR molecular detection, viral genome sequencing

## Abstract

**Simple Summary:**

Severe acute respiratory syndrome coronavirus 2 (SARS-Cov-2) is responsible for Coronavirus Disease 2019 (COVID-19). The susceptibility of experimentally infected pets, such as dogs, ferrets and cats, has been described in the recent bibliography; furthermore, the exposure of pets (dogs and cats) to SARS-CoV-2-infected owners under natural conditions was also reported. Most of the cats infected or exposed to SARS-CoV-2 were reported to be mildly symptomatic, while no study has described the clinical features and diagnostic management of symptomatic cats. This work reports the case of an indoor cat that developed clinical signs of disease after being in contact with SARS-CoV-2-infected owners and focuses on the importance of implementing a multisectoral One Health approach. Even though the viral shedding from pets does not appear sufficient to infect family members or other animals, the usual precautionary measures should urgently be considered as part of a global control system, as this integrated approach encompassing both humans and pets is pivotal for the early detection of any possible viral mutation.

**Abstract:**

Despite the reported increase in SARS-CoV-2-infected pets, the description of the clinical features from natural infection and the medical follow up in symptomatic pets is still not sufficiently documented. This study reports the case of an indoor cat that displayed respiratory signs and a gastrointestinal syndrome, following the COVID-19 diagnosis of his owners. Thoracic radiographies were suggestive of bronchial pneumonia, while blood tests were indicative of a mild inflammatory process. Nasal and oropharyngeal swabs tested positive through RT-qPCR assays targeting SARS-CoV-2 genes 14 days after his owners tested positive for the virus. Nasal swabs persisted to be RT-qPCR positive after 31 days. Serology confirmed the presence of antibodies through ELISA, electrochemiluminescence analysis and plaque reduction neutralization test, recording a high antibody titre after 31 days. The cat improved after medical treatment and clinically recovered. This study suggests that exposure to SARS-CoV-2 could lead to a natural infection with bronchial pneumonia in cats along with a possible prolonged persistence of SARS-CoV-2 RNA in the upper airways, albeit at a low level. The cat developed neutralizing antibodies, reaching a high titre after 31 days. Further descriptions of SARS-CoV-2 naturally infected pets, their medical management and diagnostic findings would be useful to enhance knowledge about COVID-19 in susceptible animals.

## 1. Introduction

Severe acute respiratory syndrome coronavirus 2 (SARS-CoV-2) was first reported in Wuhan, Hubei Province, China, in December 2019, and was confirmed to have caused Coronavirus Disease (COVID-19) [1,2]. The current SARS-CoV-2 pandemic possibly originated from an animal reservoir, most likely from bats and/or pangolins [3,4,5,6,7,8]. Since its first identification, it has been demonstrated that SARS-CoV-2 can naturally and experimentally infect several animal species, including companion animals such as cats and dogs [3,9,10,11,12,13]. Human to animal transmission has been reported in domestic, peri-domestic, wild and zoo animals [3,14,15,16,17,18,19,20,21,22,23]. As a matter of fact, the association between humans and animals, including companion animals, livestock and wildlife species, raises concerns about the potential risk of SARS-CoV-2 transmission from COVID-19 human patients to animals (“reverse zoonosis”), and about the potential role that infected animals could play in perpetuating the spread of the disease [17,24,25,26]. A case of COVID-19 human-to-animal and subsequent animal-to-human transmission has been described in Danish mink workers, although further investigations are needed to define this circumstance [22,27,28]. Evidence of exposure to SARS-CoV-2 in cats and dogs from SARS-CoV-2-infected people have been reported [4,16,29,30,31]. In pets, clinical findings ranged from asymptomatic to mild respiratory or gastrointestinal symptoms [32]. It has been described that cats naturally or experimentally infected with SARS-CoV-2 are able to transmit the virus to other cats within two days after the contact, and that the shedding of the virus most likely occurs through the respiratory and gastrointestinal tract [17,33,34,35]. It could be supposed that the virus localization in the respiratory tract may vary during the clinical phase of the disease or may depend on the clinical form, the age and the presence of concomitant systemic conditions [17,35]. Experimentally, the replication of SARS-CoV-2 in the nose and throat and a consequent inflammation pathology deeper in the lower respiratory tract (massive lesions in the nasal and tracheal mucosa epithelia and lungs) was reported in young cats [9]. Differently, a recent study has reported that in sub-adult experimentally infected cats the epithelial cells of the trachea and bronchi seemed non-permissive to SARS-CoV-2 replication, even if the SARS-CoV-2 RNA detection with RT-qPCR throughout the respiratory tract tested positive, particularly in the upper airways [17]. Furthermore, a recent study has described that SARS-CoV-2 effectively replicated in the upper respiratory tract in cats, and that the virus had cleared from the lungs within 6 days post-infection, even when asymptomatic. Moreover, histopathologic examination demonstrated chronic lung sequelae in cats even a month after viral clearance (histiocytic bronchiolitis with occlusive plugs, peribronchiolar fibrosis and thickening of alveolar septa). In addition, it revealed that after initial infection with SARS-CoV-2, cats were protected from reinfection, with no virus replication in the respiratory organs and no additional lung damage [36]. Recently, Hamer et al. delivered an epidemiological assessment of natural SARS-CoV-2 infections, including virus isolation, among serially tested cats and dogs in households with confirmed human COVID-19 cases in Texas (USA) [31], investigating the presence of SARS-CoV-2 through molecular and serological analyses. No particular clinical symptoms were detected in the dogs and cats enrolled in the study. In serological screenings, the prevalence of anti-SARS-CoV-2 antibodies in cats from Germany, Italy, Croatia, France, and China ranged from 0.69% to 23.5% [16,37,38,39,40,41]. Moreover, in a recent study from Texas, reporting the investigations on 17 cats from COVID-19-affected households [31], it emerged that only 41.2% of the tested animals presented neutralizing antibodies. In most cases, virus-neutralizing antibodies were reported and viral genome sequencing did not reveal any nucleotides coding for the spike protein following human-to-animal transmission [32,40,42,43,44,45,46]. The cat population enrolled for experimental studies is normally represented by cats without underlying health conditions, differently from the pet cat population presented to veterinarians. Human patients with underlying clinical conditions, or immunocompromised humans, were shown to have a higher risk of developing severe clinical disease when infected with SARS-CoV-2 [47], and a previous report from Spain in felines suspected the contribution of comorbidities to the clinical outcome in a cat that was found to be SARS-CoV-2 RT-qPCR positive using nasal swabs while suffering from severe respiratory distress and thrombocytopenia. After the cat was euthanized and a necropsy conducted, it was diagnosed with feline hypertrophic cardiomyopathy, severe pulmonary oedema and thrombosis [48]. Moreover, a case of a cat with symptomatic SARS-CoV-2 infection while suffering from intestinal B-cell lymphoma was reported in Northern Italy [49]. Although most of the experimentally or naturally SARS-CoV-2-infected cats were reported as being asymptomatic or mildly symptomatic, in this study, we investigated the presumptive SARS-CoV-2 infection in a cat with a mild-to-severe respiratory syndrome and gastrointestinal signs infected by COVID-19-positive owners. Despite the increase in cases reporting about SARS-CoV-2-infected pets, the description of the clinical features after natural infection and medical follow up in symptomatic cases is still not well documented [50]. Providing information such as the clinical presentation, medical management and diagnostic findings would be useful to enhance knowledge about COVID-19 disease in susceptible animals.

## 2. Materials and Methods

### 2.1. Sampling

According to our estimation of the “day zero”, the owners of a 10-year-old European shorthair neutered male cat, 5.8 kg body weight, were both diagnosed positive and symptomatic for SARS-CoV-2 by the public health service.

The cat was the only pet in the household and lived exclusively indoors. He was regularly vaccinated against Feline Calicivirus (FPV), Feline Herpes Virus Type 1 (FHV-1) and Feline Panleucopenia Virus (FPV); on Day 14, he was referred to a veterinary clinic to receive medical consultation. No previous history of respiratory or gastrointestinal illness was reported. The owners revealed that the cat had started to show apathy, anorexia, cough, respiratory distress and vomiting for 7 days before veterinary examination (Day 7). His clinical condition was getting worse. The cat was not taking any medication at the time of the visit, if we exclude the monthly administration of fipronil/(S)-methoprene/eprinomectina/praziquantel (Broadline^®^ spot on for cats, Boehringer Ingelheim Vetmedica GmbH, Ingelheim/Rhein Germany) for the prevention of ecto–endo parasite infestation. The cat was subjected to clinical investigation on Days 14 and 31.

The veterinarians wore specific personal protection equipment (facial mask, gloves, face shield and gowns) to visit the cat, and followed any other measure recommended by the international guidelines to prevent the risk of infection and spread of COVID-19 disease [51,52,53,54]. Two saliva samples were self-collected by the two veterinarians on Day 31, for SARS-CoV-2 RT-qPCR analysis. A serum sample was also checked for SARS-CoV-2 serological evaluation.

The local public health service submitted the owners’ nasal swab samples collected on Day 7 to our laboratories to compare the viral RNA genomes.

### 2.2. Time of the Study

Day 0: the SARS-CoV-2 first diagnosis of the owners; first molecular test by public health laboratory services.

Day 7: onset of clinical symptoms in the cat; owners’ second molecular test by public health laboratory services.

Day 14: first clinic, radiographic (thoracic radiographies) and laboratory investigations; first samples setting: serum, blood in K3-EDTA, nasal (N), oropharyngeal (OP) and rectal (R) swabs.

Day 31: second clinic, radiographic (thoracic radiographies) and laboratory investigations; second samples setting: serum, blood in K3-EDTA, nasal (N), oropharyngeal (OP) and rectal (R) swabs. Saliva and serum samples collected from the two veterinarians.

### 2.3. Molecular Investigation: Nucleic Acid Extraction and Qualitative Real-Time Rt-Pcr Analyses

Swabs collected from the cat on Days 14 and 31 were screened by molecular protocols for the presence of viral pathogens: N and OP swabs were tested for FHV-1, FCV and SARS-CoV-2, while R swabs were analysed for FPV [55,56,57,58], Feline Coronavirus (FCoV) (VetMAX™ FIP Dual IPC Kit, Laboratoire Service International, Lissieu, France, Applied Biosystems by Thermo Fisher Scientific, Waltham, MA, USA) and SARS-CoV-2. Nucleic acids from the N, OP and R swabs were extracted on the KingFisherTM Flex Purification System (Thermo Fisher Scientific) using the MagMAXTM Pathogen RNA/DNA kit (Applied Biosystems, by Thermo Fisher Scientific), according to the low-cell-content sample suggested by the manufacturer’s instructions. RNAs were subjected to the SARS-CoV-2 RT-qPCR protocol [59], targeting fragments of the E, N and RdRP genes on a CFX 96 Deep Well Real time PCR system (Bio-Rad Laboratories, Inc., Singapore). A universal heterologous control RNA, referred to as ‘Intype IC-RNA’ (Indical Bioscience GmbH, Leipzig, Germany), was added to each sample in the extraction step with a ratio of 1:10 of the total elution volume and amplified by using the primers and probes as per Hoffman et al. [60], in order to check the efficiency of the RNA extraction and validate each negative result. Negative and positive controls were included in each run. The Ct values equal or superior to 40.0 were considered negative. Results were generated with Bio-Rad CFX Maestro 1.1 software (Bio-Rad Laboratories).

Molecular tests on the saliva samples of the two veterinarians were performed using the same method of the RNA extraction and RT-qPCR protocol [59].

A RT-qPCR ssRNA was performed on an EDTA blood sample to exclude an ongoing Feline Leukemia Virus (FeLV) infection [55,56,57,58].

### 2.4. Serological Investigation

**Figure 1:**
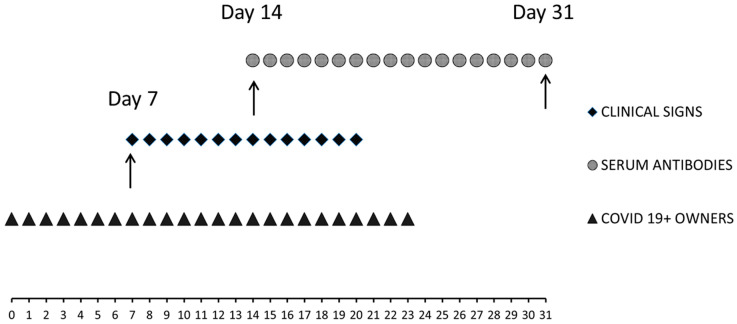
Schematic representation of the clinical case events. The cat started presenting respiratory and gastrointestinal signs on Day 7. On Day 14 occurred the first veterinary examination (sample setting and thoracic radiographies) and the beginning of medical treatment administration. On Day 21, the cat no longer presented clinical symptoms. On Day 31, the second veterinary consult (sample setting and chest radiographies) was carried out. Specific serum antibodies were detected starting from Day 14 (ELISA, ECLIA and PRNT).

The cat’s specific serological response against SARS-CoV-2 was investigated at Days 14 and 31 (see Figure 1) by means of two ELISA commercial kits, an electrochemiluminescence immunoassay (ECLIA) and plaque reduction neutralization test (PRNT).

Feline Immunodeficiency Virus (FIV) was ruled out performing an immunochromatographic commercial KIT (SNAP FIV/FeLV Combo Test, IDEXX Europe, Hoofddorp, The Netherlands) on the cat’s serum.

The serological response against SARS-CoV-2 of the two veterinarians following the clinical case was investigated only on Day 31, through an ELISA commercial kit (ID.vet Innovative diagnostics, Grabels, France), ECLIA and PRNT.

### 2.5. Electrochemiluminescence Immunoassay (ECLIA)

For the in vitro quantitative determination of antibodies (including IgG) to the SARS-CoV-2 spike (S) protein, the receptor-binding domain (RBD) in the serum of the Elecsys^®^ Anti-SARS-CoV-2 S immunoassay (Roche Diagnostics International AG, Rotkreuz, Switzerland) on a Cobas e601 analyser was used (Roche Diagnostics International AG, Rotkreuz, Switzerland). The assay is a one-step double antigen sandwich assay. A result of ≥0.8 U/mL has to be considered as reactive. Analytical performances of the method were evaluated according to the CLSI EP15-A3 protocol [61]. The test has been developed for human testing, but the double-antigen method is species-independent.

### 2.6. Elisa

ID Screen^®^—SARS-CoV-2 Double Antigen, Grabels, France ID.vet Innovative diagnostics (ELISA KIT 1) detects antibodies against the nucleocapsid (N) protein. Following the manufacturer’s instructions, we evaluated the ratio between the optical density (OD) of the sample (S) and OD of the positive control (P), as the SP% value. The sample is considered negative with SP% ≤ 50%, positive with SP% ≥ 60% and doubtful when between them. The test is validated for multi-species use, as the double-antigen method is species-independent.

The ERADIKIT^TM^ COVID-19 multi-species and total Ig, IN3 Diagnostic kit, Torino Italia (ELISA KIT 2), is an indirect ELISA for total IgG anti SARS-CoV-2. Following the manufacturer’s instructions, the sample is considered negative with an SP% < 20% and positive with an SP% ≥ 20%. The test is validated for multi-species use.

### 2.7. Plaque Reduction Neutralization Test (Prnt)

PRNT assays were performed in a Biosafety Level 3 laboratory using a SARS-CoV-2 isolate, as previously described [62]. In brief, serum samples were heat-inactivated (56 °C for 30 min) and 2-fold diluted in Dulbecco modified Eagle medium (DMEM). Serum dilutions were mixed with an equal volume (1:1) of a virus solution containing approximately 25 focus-forming units (FFUs) of SARS-CoV-2 and incubated for 1 h at 37 °C in a 5% CO_2_ incubator. Fifty microliters of the virus–serum mixtures were added to the confluent monolayers of Vero E6 cells, in 96-wells plates and incubated for 1 h at 37 °C, in a 5% CO_2_ incubator to allow for the infection of the cells. A total of 100 µL of an overlay solution made of minimum essential medium (MEM) with 2% foetal bovine serum (FBS, Sigma, Saint Louis, MO, USA), penicillin (100 U/mL, Sigma, Saint Louis, MO, US), streptomycin (100 U/mL, Sigma, Saint Louis, MO, US) and 0.8% carboxy methyl cellulose (CMC, Sigma, Saint Louis, MO, USA) were then added to each well after inoculum removal. After 26 h of incubation, the overlay was removed, and the cells were fixed with a 4% paraformaldehyde (PFA) solution. Visualization of plaques was obtained with an immunocytochemical staining method using an anti-dsRNA mouse monoclonal antibody (J2, 1:10,000; Scicons, Sziràk, Hungary) for 1 h, followed by 1 h incubation with peroxidase-labelled goat anti-mouse antibodies (1:1000; Jackson ImmunoResearch Inc., West Grove, PA, USA) and a 7 min incubation with the True Blue (KPL, Gaithersburg, MD, USA) peroxidase substrate. FFUs were counted after acquisition of pictures on a flatbed scanner. The neutralization titre was defined as the reciprocal of the highest dilution resulting in a reduction of the control plaque count >50% (PRNT_50_).

### 2.8. Sequencing Analysis

Complete genome sequencing was performed on the RNAs extracted from the cat’s OP swab sampled on Day 14 and from the nasal swabs of the two owners sampled on Day 7, using an Illumina MiSeq platform (Illumina, San Diego, CA, USA) and an in-house protocol for target amplification. After trimming and filtering for quality, reads were aligned against the reference genome (GenBank: NC_045512.2) using BWA-mem [63,64]. The sequence was deposited in GISAID under accession number EPI_ISL_962892. The virus lineage was assigned according to the PANGOLIN application, (https://pangolin.cog-uk.io/, Rambaut et al., 2020) (accessed on 23 December 2020) [65,66].

### 2.9. Clinical Procedures

After physical examination, the cat was sedated with medetomidine (Domitor^®^, ZOETIS Italia S.r.l., Rome, Italy) and propofol (PropoVet^®^, ZOETIS Italia S.r.l., Rome, Italy) via intramuscular and intravenous injections, respectively, to carry out thoracic radiographies (right lateral and dorso-ventral radiographic projections) and to collect blood (K3EDTA and serum), OP, N and R swabs. The same procedures were carried out on Day 31. All the procedures were performed for diagnostic purposes only with the owners’ informed consent.

### 2.10. Haematology and Biochemistry

The first sample of the cat’s whole blood in EDTA (Day 14) was analysed by IDEXX VetConnect^®^ PLUS Laboratories at the veterinary clinic; the second one (day 31) was processed at by with the Sysmex XN1000-V analyser (Sysmex Europe GmbH, Norderstedt, Germany).

Biochemistry was performed on the serum on Days 14 and 31 through the Cobas c501 clinical chemistry analyser with a related kit (Roche Diagnostics GmbH, Mannheim, Germany).

## 3. Results

The clinical exam performed on Day 14 revealed a normal body temperature (38.5 °C), no alteration of the oral or conjunctival mucosae membranes, mild retromandibular lymph nodes enlargement, mild dehydration status, normal abdomen examination and no cardio-circulatory alterations. The respiratory tract evaluation revealed a positive tracheal cough and an increase in pulmonary respiratory effort with moderate bronchial and pulmonary sounds. Haematology showed a mild decrease in red blood cells (RBC) and increased reticulocytes. A mild decrease in platelet count (PLT), aggregation and large platelets were detected at the blood smear examination. Serum biochemical parameters showed a mild increase in serum calcium (Ca) (2.90 mmol/L (2.26–2.73 mmol/L)) with normal total protein and albumin values. Mild hyperglycaemia (Glu) (13.9 mmml/L (3.16–8.88 mmol/L)) was observed and a mild decrease in alkaline phosphatase (ALP) (<5 U/L (6–46 U/L)) and of cholinesterase (1245 U/L (1749–2905 U/L)) were reported. Serum protein electrophoresis showed a mild increase in beta 2 protein fraction (6.1 mmol/L (3–4.7 mmol/L)). An increase in haptoglobin (99 mg/dL (18–74 mg/dL)) was described; conversely, serum amyloid A (SAA) was within the laboratory reference ranges (<0.5 µg/mL (0–9 µg/mL)) (Table 1, Table 2 and Table 3).

The OP, N and R swabs analysed through real-time RT-qPCR assay targeting the SARS-CoV-2 RNA nucleoprotein and envelope protein genes tested positive for the OP and N swabs and negative for the R swab (Table 4).

The tests for other possible viral pathogens, such as FCV (N and OP swabs), FHV-1 (N and OP swabs), FCoV and FPV (R swabs), were negative, as well as for the FIV and FeLV assays [55,56,57,58].

The cat’s serum sample tested negative using ELISA KIT 1 and positive using ELISA KIT 2. The ECLIA showed a positive value of 47.20 U/mL and the PRNT revealed a positive result with a titre of 1:5120 (Table 5).

**Figure 2:**
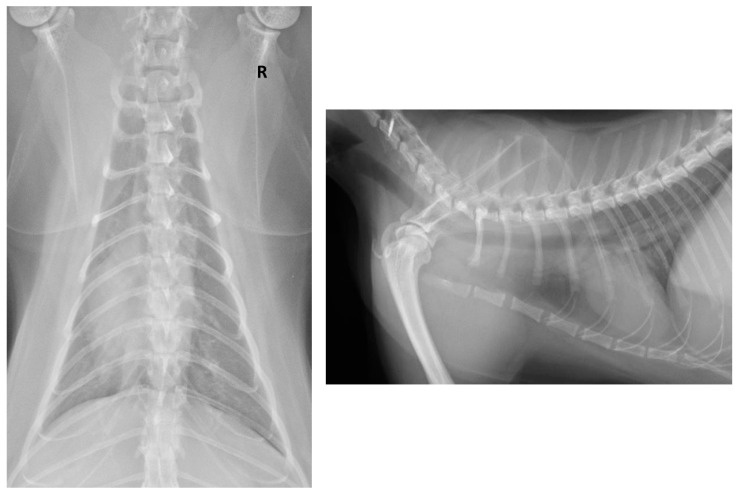
Thoracic radiographic study on Day 14: dorsoventral (DV) and right lateral radiographies (RL). The radiologic findings show a mild-to-severe diffuse bronchial pattern and a mild diffuse interstitial lung pattern. R = right side.

The thoracic radiographies revealed a mild-to-severe bronchial pattern and a diffuse interstitial lung pattern (Figure 2).

The clinical diagnosis was bronchial pneumonia potentially due to the SARS-CoV-2 viral infection. Because of the complex respiratory clinical condition, the cat received amoxicillin (Vetrimoxin^®^ Paste, Ceva Salute Animale, Agrate Brianza, Italy) at 10 mg/kg orally twice a day to prevent possible pulmonary bacterial complications and prednisone (Prednicortone^®^, Dechra, Torino, Italy) 1 mg/Kg orally once a day for 10 days. At the end of the 10 day period, the dose of prednisone was progressively decreased until it was totally suspended on Day 15 of the medical treatment [67]. The clinical status of the cat started to improve on the 3rd day of treatment, and a week later the cat no longer presented any respiratory or gastrointestinal symptoms. Fifteen days after the first examination, the veterinarian evaluated the clinical status of the cat. At this time, both owners tested negative for SARS-CoV-2 by the public health services laboratories.

The results of the second set of samples’ blood tests (Day 31), thoracic radiographies and N, PO and R swabs are described in Figure 1.

At this time, the owners reported the cat showed no clinical signs. The physical examination was unremarkable. The thorax radiographies showed a normal lung pattern and very low bronchial aspect. The haematology reported a low increase in PLT; aggregation and large platelets were detected at the blood smear examination under optic microscopy. The biochemical analysis reported mild hyperglycaemia (Glu) (11.7 mmml/L (3.16–8.88 mmol/L)) and a low increase in alanine aminotransferase (ALT) (77 U/L (19–71 U/L)) was reported. Serum protein electrophoresis showed a mild increase in the alpha 2 protein fraction (13.4 mmol/L [5.6–10.6 mmol/L]) (Table 1, Table 2 and Table 3). The RT-qPCR assay performed on the OP, N and R swabs gave a negative result for the OP and R swabs, while the N swab turned out to be slightly positive. The serology for SARS-CoV-2 tested positive by both ELISA kits. Furthermore, a relevant increase in production of SARS-CoV-2 IgG antibodies was reported through the ECLIA assay (1598 U/mL). The PRNT confirmed that the cat had developed neutralizing antibodies against SARS-CoV-2 (1:2560) (Table 4 and Table 5).

The cat had completely recovered after 15 days (Day 31) and did not show any residual respiratory or gastrointestinal signs.

The veterinarians tested negative for SARS-CoV-2.

A complete genome sequencing was performed on the RNA extracted from the cat’s OP. The virus was assigned to the lineage B.1.177 [65,66], a common lineage in humans in Italy (sequence data available in GISAID as of 14 January 2021). Furthermore, none of the mutations that have occurred to date in the SARS-CoV-2 spike following human-to-animal transmission has been identified. Only the partial genome was obtained from the owners’ samples, most probably as a consequence of the low viral titre at the time of the sampling. However, the data generated allowed to cover 93% of the genome, which was sufficient to confirm the clustering of the sequence within the same lineage.

## 4. Discussion

Concomitantly with the outbreaks of COVID-19 disease, a relevant number of SARS-CoV-2 naturally infected cats have been reported by the Office International des Epizooties (OIE) in different geographic areas (United States, Latin America, Spain, Switzerland, the Netherlands, Germany, France and China) [68,69,70]. Several studies have recently reported natural human-to-pet SARS-CoV-2 transmission in close contact conditions (COVID-19-positive households). The reported clinical features were classified as asymptomatic or mildly symptomatic (lethargy, sneezing); thus, the prevalence of SARS-CoV-2 infection in cats, especially for the asymptomatic cases, may be underestimated [31]. The present study describes a natural human-to-cat SARS-CoV-2 transmission. The cat showed respiratory and gastrointestinal syndromes, even if we may not exclude a mild–low concurrent bacterial infection. This may justify the response to the medical treatment, but it could also represent the natural course of the COVID-19 viral infection. Thoracic radiographic alterations suggestive of bronchial pneumonia were observed. Such abnormalities likely indicate a bronchial inflammatory process in association with pulmonary inflammatory infiltrate or fibrotic tissue. Recently, possible prolonged and persistent pulmonary sequelae in SARS-CoV-2 infected cats have been reported [36]. Even if the pathogenic events and consequences of SARS-CoV-2 in cats have yet to be comprehensively recorded, it has been observed that cats seem to possess a SARS-CoV-2 pulmonary receptor binding model similar to the one in humans. This would explain the susceptibility of felines to SARS-CoV-2 and may describe both the development of the respiratory syndrome and the chest radiographic abnormalities [71,72]. The mild regenerative normocytic normochromic anaemia (mild decrease in RBC) and increased reticulocytes, reported on Day 14, may be consistent with an ongoing inflammatory process [73]. The mild hyperglycaemia detected, similar in both the Day 14 and Day 31 samples, was probably due to the administration of the sedative drugs and to the stressful overall situation [74,75]. The serum electrophoresis increase in the beta 2 protein fraction was probably connected with the clinical inflammatory status and the immunologic response, such as antibodies production, particularly the immunoglobulin complement fraction [76]. The mild decrease in ALP and in cholinesterase was likely due to an impaired liver function consequent to the gastrointestinal symptoms, such as vomit and loss of appetite [77,78]. The increase in haptoglobin reflected an ongoing inflammatory process [76,79], whereas the Feline SAA was possibly within the laboratory reference ranges considering that Feline SAA concentrations increase early during inflammation, usually in concomitance with other clinical signs (e.g., fever) or increase in the haematological parameter, such as leukocytosis [80]. At the time of the second sampling set (Day 31), a mild increase in PLT value was found, which may be related both to the inflammatory process and, more likely, to the PLT activation and aggregation during the blood sampling (pseudothrombocytopenia) [81,82]. A low increase in ALT and in the serum protein electrophoresis alpha 2 protein fraction would be related both to the medical treatment and the systemic inflammation [77,83]. The cat showed clinical signs 7 days after the owners had been confirmed positive for COVID-19 and were symptomatic themselves (Day 7). In this study, the cat showed a clinical recovery and developed neutralizing antibodies from Day 14, reaching a high antibody titre after 31 days. Such a titre may be considered protective for a reinfection, as previously reported [16,32,35,36,39,41,84]. The RT-qPCR assay performed on OP, N and R swabs resulted positive for the N and OP swabs on Day 14, negative for the OP swab and weak positive for the N swab on Day 31, respectively. The R swabs both tested negative, suggesting a rapid clearance of the virus from the intestinal tract. In experimental studies, cats stop shedding the virus within 10 days [3,9,35], while more recent studies reported that the SARS-CoV-2 experimentally infected cats may have a prolonged period of oral and nasal viral shedding not accompanied by clinical signs, and are capable of direct contact transmission to other cats [35,42]. In agreement with a recent paper [31], we report a prolonged persistence of SARS-CoV-2 infection (N swabs positive in RT-qPCR on Day 31), although the presence of the viral agent is low and presumably not sufficient to infect other susceptible subjects. Although we reported a very low positivity in the last RT-qPCR assay (N swab), it was not possible to perform further diagnostic procedures as the owners denied their consent.

As only limited information is available so far on the potential viral shedding routes, it would be beneficial to investigate for SARS-CoV-2 in those cats referred to veterinary clinics for respiratory or gastrointestinal symptoms developed after or concomitantly to their COVID-19-positive owners. Furthermore, SARS-CoV-2 diagnostics in cats living in particular situations and environments, such as dense housing conditions, close contact with elderly people, cat rescue or breeding centres, would be crucial. The recently emerged variants (B.1.1.7. and B.1.351) may have a fitness advantage associated with mutations in the spike protein, which are suspected to lead to an increase in human-to-human transmissibility and more effective replication [65,85]. Possible changes in the susceptibility of animals in the context of these new variants should be evaluated [86,87]. Fortunately, with reference to this specific case, SARS-CoV-2 genomic mutations which may possibly be involved in the animal-to-human transmission have not been reported so far, although the diagnostic and clinical surveillance, as well teaching how to implement preventions measures, are issues of utmost importance.

## 5. Conclusions

The diagnosis of SARS-CoV-2 infection in pets such as cats would be extremely important (i) to provide appropriate veterinary care for the infected animals; (ii) to guarantee adequate protection of veterinary staff and pet owners; and (iii) to apply quarantine measures to prevent transmission between pets, people and potentially susceptible animals. Even though the viral shedding from pets does not appear sufficient to infect other family members or other animals, the usual precautionary measures should urgently be considered as part of the global control efforts and One Health approach. There is currently no evidence that cats play a significant role in human infection and in the spread of the virus to humans. The recently emerged variants (B.1.1.7. and B.1.351) may raise concerns about the possible involvement of susceptible species in new mutations [70] and about the chance of severe clinical signs in animals [87]. Thus, reverse zoonosis is possible if infected owners expose their pets to the virus, particularly during the acute phase of the infection. It is important that pet owners are educated to adopt the precautionary measures to avoid human-to-cat SARS-CoV-2 transmission [88]. Preventing interspecies transfer of an emergent pathogen is essential to decrease the risk of emerging mutations that could affect the transmissibility or effectiveness of the countermeasures, and is also needed to safeguard pet welfare and discourage animal abandonment.

## Figures and Tables

**Table 1:** Haematology performed from K3EDTA blood samples on Days 14 and 31, respectively. The first sample was analysed by IDEXX VetConnect^®^ PLUS Laboratories, and the second (Day 31) was performed at the SCS3 Laboratory Medicine of the IZSVE with the Sysmex XN1000-V analyser (Sysmex Europe GmbH, Norderstedt, Germany).

Haematology	IDEXX VetConnect^®^ PLUS	Laboratory Medicine (IZSVE)
	Day 14	Day 31
RBC (M/μL)	6.9 (7.1–11.5)	7.69 (5.1–10)
Hgb (g/dL)	9.4 (10.3–16.2)	11.1 (8–15)
Hct (%)	32.6 (28.2–52.7)	31.4 (30.0–45.0)
MCV (fL)	47.2 (39–56)	40.8 (39–55)
MCH (pg)	13.6 (12.6–16.5 pg)	14.4 (13.0–17.0)
MCHC (g/dL)	28.8 (28.5–37.8)	35.4 (30.0–36.0)
Reticulocytes (K/μL)	8.3 *	-
RDW (%)	-	17.3 (16.4–21.7)
PLT (K/μL)	133(155–641)	430 (100–400)
MPV (fL)	-	13.3 (8.1–15.4)
WBC (K/μL)	7.1 (3.9–19)	7.24 K/μL (5–19)
NEUT (K/μL)	4.52 (2.62–15.17)	3.79 (1.80–14.80)
LYMPH (K/μL)	1.98 (0.85–5.85)	3.29 (1.10–8.60)
MONO (K/μL)	0.28 (0.04–0.53)	0.09 (0.05–0.80)
EO (K/μL)	0.28 (0.09–2.18)	0.06 (0.05–2.30)
BASO (K/μL)	0 (0.09–2.18)	0.01 (0.00–0.80)

* Reticulocytes/uL < 50,000 is normal in case of a non-anaemic patient; <50,000 is inadequate in case of an anaemic patient; 50,000–75,000 is mild regeneration; 75,000–175,000 is moderate regeneration; and >175,000 is marked regeneration.

**Table 2:** Biochemistry performed on Days 14 and 31, respectively. Analyses were performed on serum through the Cobas c501 clinical chemistry analyser with a related kit (Roche Diagnostics International AG, Rotkreuz, Switzerland) at the SCS3 Laboratory Medicine of the IZSVE.

BIOCHEMISTRY	Day 14	Day 31	Reference Values
Haptoglobin ^1^	99	18	18–74 mg/dL
Serum Amyloid A ^2^	<5.0	<5.0	0–9 μg/mL
Total Proteins	68 g/L	72 g/L	62–80 g/L
Albumin	38 g/L	40 g/L	30–47 g/L
Globuline	30 g/L	32 g/L	22–47 g/L
Ratio A/G	1.28	1.40	1.07–1.87
Urea Nitrogen	10.0	7.3	4.8–12.6 mmol/L
Creatinine	150	122	66–178 μmol/L
Glucose	13.9	11.7	3.2–8.9 mmol/L
Cholesterol	4.37	4.79	1.35–6.09 mmol/L
Triglycerides	0.58	4.21	0–2.48 mmol/L
Total Bilirubine	<2.5	<2.5	0–8.55 μmol/L
Direct Birubine	<1.5	<1.5	0–2.56 μmol/L
Unconj Bilirubine	0	0	0–6.5 μmol/L
AST	15	29	0–61 U/L
ALT	27	77	19–71 U/L
ALP	<5	17	6–46 U/L
GGT	<3	<3	1–5 U/L
Cholinesterase	1245	1781	1749–2905 U/L
CK	11	141	0–305 U/L
Calcium	2.90	2.38	2.26–2.73 mmol/L
Phosphorus	1.43	1.03	0.94–1.98 mmol/L
Magnesium	0.92	0.88	0.79–1.07 mmol/L
Sodium	152	151	141–168 mmol/L
Potassium	4.37	4.29	3.55–5.15 mmol/L
Chlorine	114	112	103–126 mmol/L
Iron	74 μg/dL	82	68–215 μg/dL
Uibc	168 μg/dL	*	105–205 μg/dL
Tibc	242 μg/dL	*	222–423 μg/dL
Saturated Transferrin	30.6%	*	20–56%

* Sample insufficient. Analysis not performed. AST (serum glutamic oxaloacetic transaminase), ALT (serum glutamic pyruvic transaminase), ALP (alkaline phosphatase), ALT (gamma glutamil transferase), CK (creatin kinase). ^1^ Haptoglobin was measured with a Tridelta PHASE Haptoglobin Assay (Tridelta Development Limited, Maynooth, County Kildare, Ireland) on a Cobas c501 analyser. ^2^ Serum amyloid A was measured with a multispecies VET-SAA kit (Eiken Chemical Co Ltd., Tokyo, Japan) on a Cobas c501 analyser.

**Table 3:** Serum protein electrophoresis (Minicap, Sebia Italia S.r.l., Firenze, Italy) on Days 14 and 31, respectively. A mild increase in alpha 2 globulin and beta 1 globulin on Day 14 was reported, consistent with the inflammatory response and the antibodies production.

Serum Protein Electrophoresis	Day 14	Day 31	Reference Values
Albumin (%)	56.2	58.4	52.4–66.2
Alpha 1 (%)	1.3	1.9	0.8–1.9
Alpha 2 (%)	15.6	18.6	7.4–15.4
Beta 1 (%)	9.0	5.7	4.5–6.2
Beta 2 (%)	5.5	5.8	4.5–8
Gamma (%)	12.4	9.6	8.5–24.2
Albumin (g/L)	38.2	42.0	35.7–48.7
Alpha 1 (g/L)	0.9	1.4	0.6–1.3
Alpha 2 (g/L)	10.6	13.4	5.6–10.6
Beta 1 (g/L)	6.1	4.1	3–4.7
Beta 2 (g/L)	3.7	4.2	3.2–5.8
Gamma (g/L)	8.4	6.9	5.1–18.3
A/G Ratio	1.28	1.40	1.07–1.87

**Table 4:** Results of the real-time RT-qPCR performed on Days 14 and 31, using oropharyngeal, nasal and rectal swabs collected at Days 14 and 31, targeting the SARS-CoV-2 RNA.

Day 14					Day 31			
-	Ct Values			Conclusive Laboratory Diagnosis	Ct Values			Conclusive Laboratory Diagnosis
Swab	E Gene	N Gene	RdRp Gene	-	E Gene	N Gene	RdRp Gene	-
OP	30.14	36.38	39.60	Positive	n.d.	n.d,	n.d,	Negative
N	27.83	34.47	36.00	Positive	36.00	n.d.	n.d.	Positive
R	n.d.	n.d.	n.d.	Negative	n.d.	n.d.	n.d.	Negative

OP—oropharyngeal swab; N—nasal swab; R—rectal swab; n.d.—not detected; N gene—nucleocapsid protein; E gene—envelope protein gene; RdRp gene—RNA-dependent RNA polymerase gene.

**Table 5:** Serological assays performed at different times: first cat’s blood sample collected 7 days after the beginning of the respiratory and gastrointestinal symptoms on Days 14 and 31.

SEROLOGY SARS-CoV-2	Day 14	Day 31	Reference Ranges
ELISA KIT 1	NEGATIVE	POSITIVE (68%)	Cut-off ≥ 60%
ELISA KIT 2	POSITIVE (33.6%)	POSITIVE (20.8%)	Cut-off ≥ 20%
ECLIA	47.20 U/mL	1598 U/mL	POSITIVE ≥ 0.8 U/mL
PNRT	1:5120	1:2560	<1:10

ELISA KIT 1, ELISA KIT 2, SARS-CoV-2 ECLIA (electrochemiluminescence: Elecsys anti SARS-CoV-2 S double antigen assay for the detection of IgG antibodies against coronavirus RBD spike protein, Roche Diagnostics), and PRNT (plaque reduction neutralization test).

## Data Availability

The data presented in this study are available in the article.
